# Formation of the synaptonemal complex in a gynogenetic allodiploid hybrid fish

**DOI:** 10.3389/fgene.2023.998775

**Published:** 2023-02-27

**Authors:** Jing Wang, Wen Wang, Jihong Li, Yirui Zhang, Kaikun Luo, Linmei Han, Caixia Xiang, Mingli Chai, Ziye Luo, Rurong Zhao, Shaojun Liu

**Affiliations:** ^1^ State Key Laboratory of Developmental Biology of Freshwater Fish, College of Life Sciences, Hunan Normal University, Changsha, China

**Keywords:** gynogenesis allodiploid hybrids (GDH), meiosis, homologous chromosome, synaptonemal complex, unreduced gametes

## Abstract

**Introduction:** The correct pairing and separation of homologous chromosomes during meiosis is crucial to ensure both genetic stability and genetic diversity within species. In allodiploid organisms, synapsis often fails, leading to sterility. However, a gynogenetic allodiploid hybrid clone line (GDH), derived by crossing red crucian carp (*Carassius auratus* ♀) and common carp (*Cyprinus carpio* ♂), stably produces diploid eggs. Because the GDH line carries 100 chromosomes with 50 chromosomes from the red crucian carp (RCC; ♀, 2n = 2x = 100) and 50 chromosomes from the common carp (CC; *C. carpio* L., ♂, 2n = 2x = 100), it is interesting to study the mechanisms of homologous chromosome pairing during meiosis in GDH individuals.

**Methods:** By using fluorescence *in situ* hybridization (FISH) with a probe specific to the red crucian carp to label homologous chromosomes, we identified the synaptonemal complex *via* immunofluorescence assay of synaptonemal complex protein 3 (SCP3).

**Results:** FISH results indicated that, during early ovarian development, the GDH oogonium had two sets of chromosomes with only one set from *Carassius auratus,* leading to the failure formation of normal bivalents and the subsequently blocking of meiosis. This inhibition lasted at least 5 months. After this long period of inhibition, pairs of germ cells fused, doubling the chromosomes such that the oocyte contained two sets of chromosomes from each parent. After chromosome doubling at 10 months old, homologous chromosomes and the synaptonemal complex were identified.

**Discussion:** Causally, meiosis proceeded normally and eventually formed diploid germ cells. These results further clarify the mechanisms by which meiosis proceeds in hybrids.

## Introduction

In sexually reproducing organisms, haploid germ cells (eggs or sperm), with half the number of chromosomes of the parental somatic cells, are produced by meiosis. During meiosis, diploid cells undergo DNA replication, followed by two rounds of cell division. Meiosis is divided into two phases: meiosis I and meiosis II. The significant difference between prophase I and prophase II is the formation of tetrads in prophase I (Bolcun-Filas and Handel, 2018). In prophase I, a large number of programmed DNA double strand breaks are produced through the activity of the *spo11* gene, and base repair occurs basing on homologous recombination of homologous chromosomes, which sequentially undergoes recognition, pairing, synapsis, and recombination (Zickler and Kleckner, 2015). Several meiosis-related genes are involved in these processes. *Rad50* and *dmc1* are important recombinases in meiosis, and Synaptonemal Complex Protein 3 (SCP3) is critical for the formation of the synaptonemal complex (Bishop et al., 1992; Iwai et al., 2006). In addition, previous studies have shown that the association protein MLH1 and the recombinant protein MSH5 play crucial roles in the association and recombination of homologous chromosomes (Edelmann et al., 1996; Snowden et al., 2004). MLH1 presents on the chromosomes during the pachytene phase, stabilizes the exchange sites and favors the pairing of homologous chromosomes. MLH1 can thus serve as a marker protein for homologous recombination (Edelmann et al., 1996; Feitsma et al., 2007). MSH5 is essential for the stabilization of Holliday junction intermediates, which in turn promote recombination (Snowden et al., 2004). In order to produce normal haploid gametes and maintain genetic diversity, these processes must complete successfully (Wells et al., 2020). Thus, both normal homologous chromosome pairing and the normal expression of these genes are required for successful meiosis.

Disfunction in the synapsis and recombination of homologous chromosomes leads to the activation of the pachytene checkpoint (Roeder and Bailis, 2000). The pachytene checkpoint interrupts the cell cycle in cells and activates a series of factors downstream to repair the damaged processes (de Cárcer et al., 2007). Thus, the pachytene checkpoint can be considered as a meiotic surveillance system. Under this surveillance system, meiosis is allowed to proceed once all the chromosomes have completed pairing, and the meiotic process is halted if pairing is not completed. Thus, the pachytene checkpoint can inhibit the production of defective gametes, such as aneuploid gametes, by averting chromosome missegregation and detecting aberrant meiotic products (Roeder and Bailis, 2000; Li et al., 2009).

Hybridization is an important breeding method, but the hybrid offspring are often sterile, primarily due to the abnormal synapsis of homologous chromosomes (Bhattacharyya et al., 2013; Flachs et al., 2014). However, we successfully obtained hybrids that are fertile and thus seem to have overcome normal reproductive barriers (Liu et al., 2007; Wang et al., 2016). The fertile allotetraploid hybrids, which produce diploid gametes, were generated *via* the distant hybridization of the red crucian carp (RCC; *Cyprinus auratus* red var., ♀, 2n = 2x = 100) and the common carp (CC; *C. carpio* L., ♂, 2n = 2x = 100). After activation with UV-irradiated sperm, the diploid eggs produced by this allotetraploid hybrid developed into gynogenetic allodiploid hybrids that also produced diploid eggs (Wang et al., 2016). This gynogenetic allodiploid hybrid clonal line (GDH, n = 2x = 100) reaches sexual maturity at 2 years old and stably produces allodiploid eggs (Wang et al., 2016). Previous studies have shown that the first and second pole body expulsions are normal during the formation of unreduced gametes (Zhang et al., 2005). The formation of the diploid egg is associated with the doubling of genetic material during early stage of gonad development *via* germ cell fusion (Wang et al., 2016).

Typically, gametogenesis, which requires normal meiosis, synapsis, and recombination, is blocked in hybrids due to the absence of homologous chromosomes, resulting in the sterility of allodiploid hybrids (Huang et al., 2018). However, the allodiploid GDH hybrids are fertile and produce diploid eggs (Wang et al., 2016). It is unknown whether synapsis and recombinations happen normally in GDH.

In this paper, we aimed to characterize chromosome behavior during meiosis prophase I in GDH to clarify the mechanisms underlying the formation of fertility in these allodiploid hybrids. We focused on synapsis and recombination during prophase I in GDH. Our results will help to clarify the mechanisms underlying the formation of unreduced gametes in fish, as well as the regulatory pathways associated with the pachytene checkpoint and the reproductive characteristics of hybrid progeny.

## Materials and methods

### Ethics statement

The first generation of gynogenetic offspring (GDH_1_) (1997) was obtained by artificial gynogenesis from the eggs of AT that were activated with ultraviolet (UV)-treated sterilized sperm of blunt snout bream without treatment for doubling the chromosomes. The GDH line carries 50 chromosomes from the red crucian carp (RCC; ♀, 2n = 2x = 100) and 50 chromosomes from the common carp (CC; *C. carpio* L., ♂, 2n = 2x = 100). The GDH fish used in this experiment is the 12th generation (GDH_12_), which was obtained by artificial gynogenesis from the diploid eggs of GDH_11_ that were activated with ultraviolet (UV)-treated sterilized sperm of blunt snout bream without treatment for doubling the chromosomes. All GDH fish were cultured in ponds at the State Key Laboratory of Developmental Biology of Freshwater Fish (Changsha, China) and fed with artificial feed. All experiments were approved by the Animal Care Committee of Hunan Normal University (Changsha, China) and followed the University’s guidelines for the care of experimental laboratory animals (GB/T 35,823/2018). Fish were deeply anesthetized with 100 mg/L MS-222 (Sigma-Aldrich, St Louis, MO, United States) before dissection.

### Observation of microstructure and ultrastructure of the germ cell

To observe germ cell structure, we randomly selected six GDH individuals every month between 2 and 12 months of age. The ovary was fixed in 4% paraformaldehyde (PFA). Paraffin-embedded ovaries were cut into sections (5 μm thick) and stained with hematoxylin and eosin. Tissue microstructure was then observed and photographed under a microscope (CKX41-32 PH, Olympus, Tokyo, Japan). Partial ovary from each individual was then fixed in 2.5% glutaraldehyde and embedded in Epon812 (Sigma-Aldrich, St. Louis, MO, United States). Ultrathin sections (60 nm) were cut. The sections were stained with uranyl acetate and lead citrate. We then observed the ultrastructure in each section using a transmission electron microscope (HT7800, Hitachi, Tokyo, Japan).

### Observation of the growth of germ cells *in vitro* and the identification of germ cells

A small piece of gonadal tissue was taken from the above GDH individuals for *in vitro* culture at 26°C under 5% CO_2_ in Dulbecco’s modified Eagle’s medium (Gibco BRL, Burlington, ON) containing 5 ng/mL bFGF (Gibco BRL), 15% FBS (Hyclone Labs Inc., Logan, UT), 100 U/mL penicillin, 100 μg/mL streptomycin, and 5% heat-inactivated RCC serum. After 72 h of culture, the living germ cells were observed under an inverted light microscope (Ti-E, Nikon, Tokyo, Japan).

The germ cells were identified using immunofluorescence. The cultured cells were fixed in 4% PFA. Then the cells were incubated at 4°C overnight with the anti-VASA antibody (1:100; ab209710, Abcam, Cambridge, MA, United States). The secondary antibody was Dylight488 anti-rabbit IgG. Images were captured using a laser scanning confocal microscope (FV1200, Olympus, Tokyo, Japan).

### Preparation of chromosome spreads and immunofluorescence of synaptonemal complexes

Chromosome spreads were prepared using head kidney cells, oogonia, and oocytes extracted during prophase I (as identified above) from ten individuals. Ten chromosome spreads were selected from each individual for subsequent analysis. First, each tissue was ground in 0.8% NaCl and the upper cell suspension was centrifuged for 1 min at 289 × *g*. Second, the cells collected by centrifugation were immersed in a hypotonic solution consisting of 0.075 M KCl for 120 min (germ cells) or 45 min (kidney cells), then fixed in a 3:1 methanol: acetic acid solution. Finally, the fixed cells were transferred to a cold slide, stained for 30 min with 4% Giemsa in phosphate buffer (pH 7.0), observed, and photographed under a light microscope (CKX41-32 PH, Olympus, Tokyo, Japan). SCP3 content was detected using immunofluorescent staining with anti-SCP3 antibodies (ab150292, Abcam, Cambridge, MA, United States) as previously described (Chen et al., 2016).

### Fluorescence *in situ* hybridization (FISH) of chromosomes

We constructed specific probe for fluorescence *in situ* hybridization (FISH) from genomic DNA of the RCC blood that specifically labeled crucian carp chromosomes: a chromosomal arm probe, using a 340-bp repeat from the 5S rDNA sequence (GenBank accession no. KM359663). The FISH probe was labeled with Dig-11-dUTP by PCR DIG Probe Synthesis Kit (Roche, Penzberg, Germany), following the manufacturer’s instructions with 50ul reaction solution contain 10 ng plasmid DNA template. The 5S rDNA sequence was amplified using the primers 5′- GCT​ATG​CCC​GAT​CTC​GTC​TGA-3′ and 5′-CAG​GTT​GGT​ATG​GCC​GTA​AGC-3′. The PCR cycling conditions were as follows: pre-denaturation at 94°C for 5 min; 30 cycles of denaturation at 94°C for 30 s, annealing at 53°C for 45 s, extension at 72°C for 1 min; a final extension at 72°C for 10 min; and finally an infinite hold at 4°C. FISH was performed as previously described (He et al., 2012). Slides were viewed and images were captured using a laser scanning confocal microscope (FV1200, Olympus, Tokyo, Japan).

### RNA isolation and real-time quantitative PCR (qPCR)

The expression patterns of four genes associated with synapsis and recombination (*spo11, scp3, rad50,* and *dmc1*) were compared among ovaries at different developmental stages using qPCR. Total RNA was isolated from 5-month-old, 10-month-old, and 12-month-old GDH ovaries (3 samples per group) using TRIzol Reagent (Invitrogen, Carlsbad, CA, United States). RNA quantity was determined using a spectrophotometer (Synergy2, Bio Tek, Burlington, VT, United States). After a treatment with RNase-free DNase (Promega, Madison, WI, United States), total RNA was reverse-transcribed to complementary DNA (cDNA) using ReverTraAce M-MLV (Toyobo, Osaka, Japan) with random primers. qPCR was carried out on a QuantStudio5 instrument (Applied Biosystems, Carlsbad, CA, United States) using PowerUp TM SYBR TM Green Master Mix (Applied Biosystems, Carlsbad, CA, United States). The reaction mixture (10 µL) contained 2.5 µL cDNA (200 ng/μL), 5 µL PowerUp TM SYBR TM Green Master Mix, 0.5 µL specific forward primers, 0.5 µL reverse primers, and 1.5 µL water. The amplification conditions were as follows: 50°C for 5 min; 95°C for 10 min; and 40 cycles of 95°C for 15 s and 60°C for 45 s. The average threshold cycle (Ct) of the target genes was calculated for each sample using the 2^−ΔΔCT^ method and normalized to *β-actin*. The primers used in this study were designed by the authors, as shown in Table 1. Data are expressed as mean ± standard error of the mean (S.E.M., n = 3). Independent-sample Student’s t-tests were performed in GraphPad Prism 7.04 (San Diego, CA) to identify significant differences between samples. We considered *p* < 0.05 statistically significant.

**TABLE 1:** Primers used for qPCR.

Gene	Sequence (5'→3′)
*spo11*	F: CTT​CCA​ATG​TTA​ATG​GGA​TCA​GA
	R: CAG​TTT​CCT​CAC​CAT​CAG​TCT​GC
*scp3*	F: AAG​ATT​CGG​TGC​GGA​CAT​C
	R: GCT​TCT​GCC​TCT​GAT​TCT​GC
*rad50*	F: TCT​CGC​CAA​CGC​AAC​TTC​C
	R: GCA​CTG​GTC​CTG​GTT​CTT​CC
*dmc1*	F: ACT​ATG​TGT​AAC​CGC​TCA​G
	R: GAT​GCT​CAC​TCG​TAT​ATG​C
*β-actin*	F: CAT​CTA​CGA​GGG​TTA​CGC​CC


### Western blot

Ovaries from 5-, 8-, and 10-month-old GDHs were lysed using Tissue or Cell Total Protein Extraction Kits (Sangon Biotech, Shanghai, China). The lysates were first separated on 12.5% sodium dodecyl sulfate polyacrylamide (SDS-PAGE) gels and then transferred onto polyvinylidene fluoride (PVDF) membranes. The separated proteins were immunoblotted with rabbit anti-MSH5 (dilution 1:1,000; PA5-66516, Invitrogen, Carlsbad, CA, United States), rabbit anti-MLH1 (dilution 1:2000; ab92312, Abcam, Cambridge, MA, United States), and mouse anti-H3 (dilution 1:100,000; GB13102-1, servicebio, Wuhan, China). After washing, the membranes were incubated with a horseradish peroxidase (HRP)-conjugated anti-rabbit secondary antibody (dilution 1:2000; ab205718, Abcam, Cambridge, MA, United States) and an HRP-conjugated anti-mouse secondary antibody (dilution 1:2000; ab205719, Abcam, Cambridge, MA, United States). Blot bands were visualized using an electrogenerated chemiluminescence (ECL) reagent (G2014, servicebio, Wuhan, China). The results were normalized using H3 as an internal control.

## Results

### Microstructural and ultrastructural characteristics of germ cells

**FIGURE 1:**
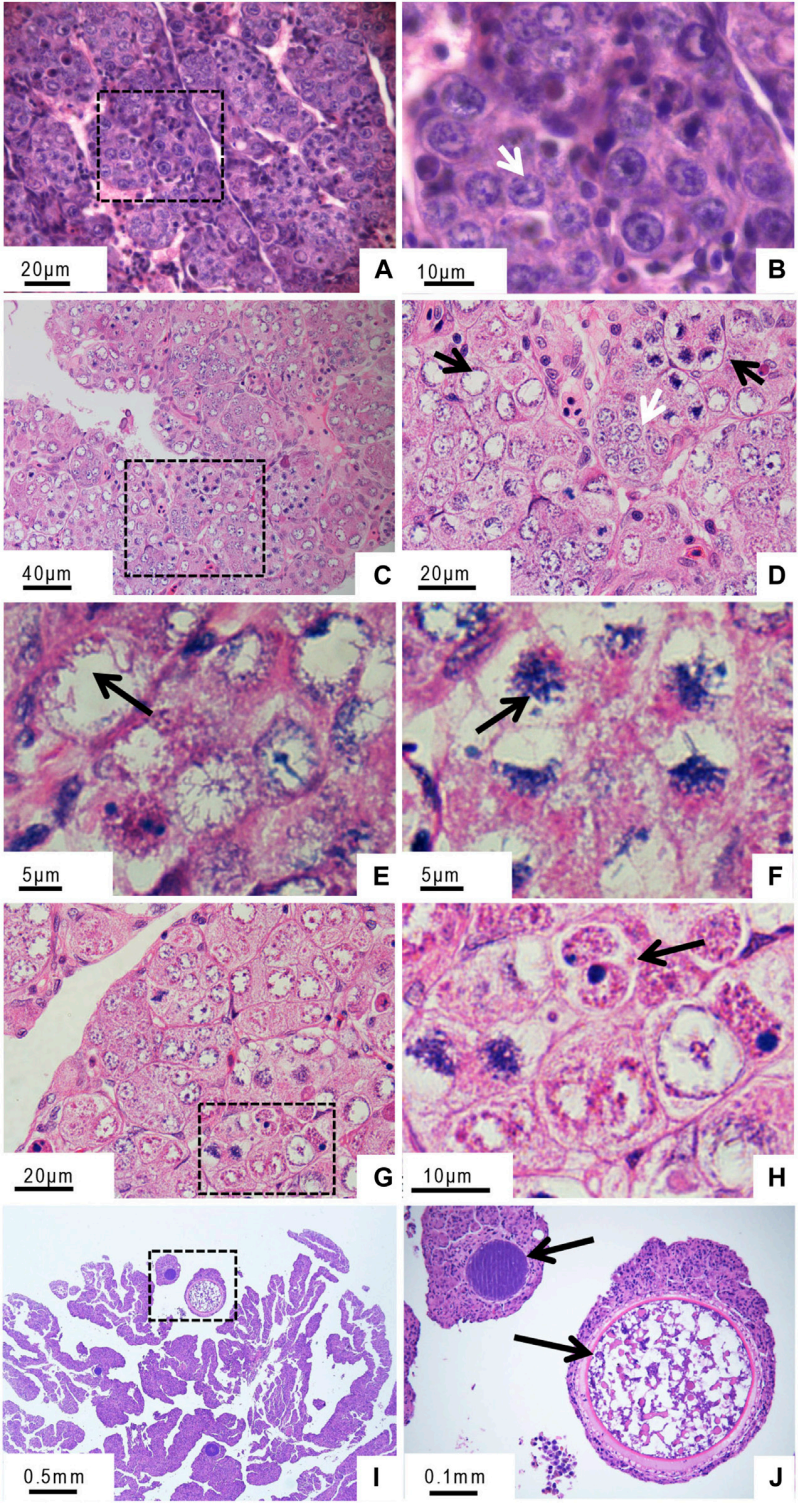
Histological microstructure of the gynogenetic allodiploid hybrid clonal line (GDH) ovary during early development **(A, B)** At 5 months old **(C–H)** At 10 months old **(I, J)** At 12 months old. Arrows in panels indicate the following features **(A, B)** Oogonia **(C, D)** Oogonia (white arrow) and oocytes (black arrow) **(E, F)** Chromosomal bouquet **(G, H)** Cell with two nuclei **(I, J)** Oocyte in the growth phase.

During early GDH development (<5-month-old), cells in the ovary were oogonia of uniform size, with densely stained round nuclei and uniform chromatin distributions (Figures 1A, B). In 10-month-old GDH ovaries, the cells were primarily oocytes, with a few oogonia (Figures 1C, D). Unlike the germ cells from the 5-month-old ovary, the nuclei of the germ cells from the 10-month-old ovary had uneven staining due to non-uniform chromatin distribution. Chromosomes were aggregated in a configuration known as the bouquet, in which the chromosomes were clustered around or at one end of the nuclear envelope (Figures 1E, F). At this stage, two nuclei were observed in some cells (Figures 1G, H). In the 12-month-old ovary, the oocytes were in the growth phase, and the cell volume was noticeably increased due to yolk accumulation (Figures 1I, J).

**FIGURE 2:**
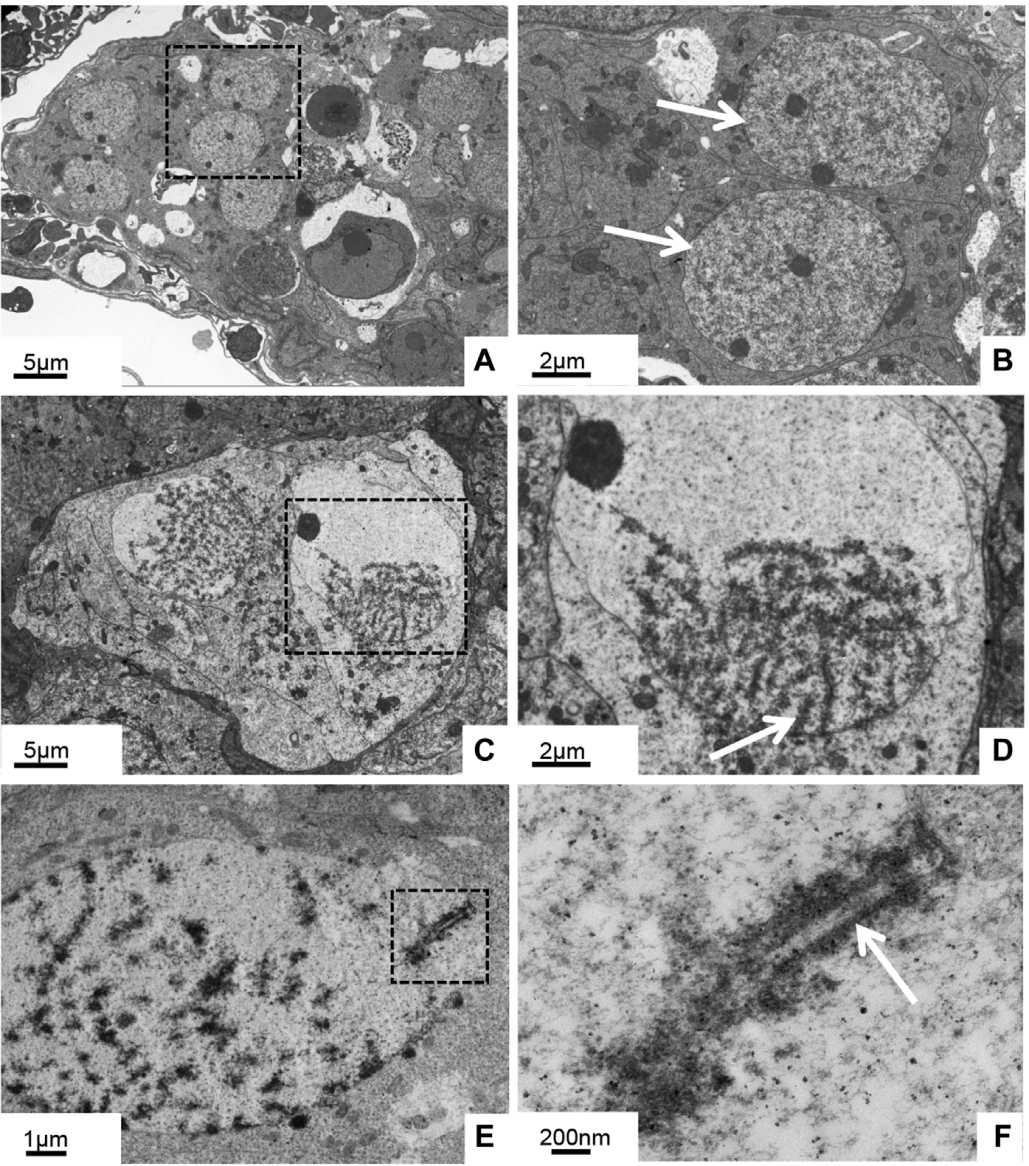
Histological ultrastructure of the gynogenetic allodiploid hybrid clonal line (GDH) ovary during early development **(A, B)** At 5 months old **(C–F)** At 10 months old **(A, B)** Oogonia with homogeneous chromatin **(C, D)** Oocytes with chromosomes clustered at one end of the nuclear membrane **(E, F)** Oocytes with synaptonemal complexes. Arrows indicate parallel segments of homologous chromosomes.

Further electron microscopic observation of the 5-month-old ovary showed that chromatin distribution was uniform in the oogonia, and the nucleoli were obvious (Figures 2A, B). In the ovaries of 10-month-old GDH, the oocyte nucleus was larger than that of the oogonia, and the chromosomes were gathered in a unipolar radial arrangement (Figure 2C). The distribution of chromosomes in the nucleus was radially diffuse, forming a flower-like shape. This stage, which is also known as the bouquet or condensate-line stage, lasts from the end of leptotene to the end of the zygotene (Harper et al., 2004). The telomeres aggregated in one main group at the nuclear envelope, while the chromosomal axes described arches toward the nuclear space (Figure 2D). Oocytes at bouquet stage were enriched at this point, obvious synaptonemal complexes were observed between chromosomes, and some segments of homologous chromosomes were arranged in parallel (Figures 2E, F).

### Germ cell proliferation and fusion

**FIGURE 3:**
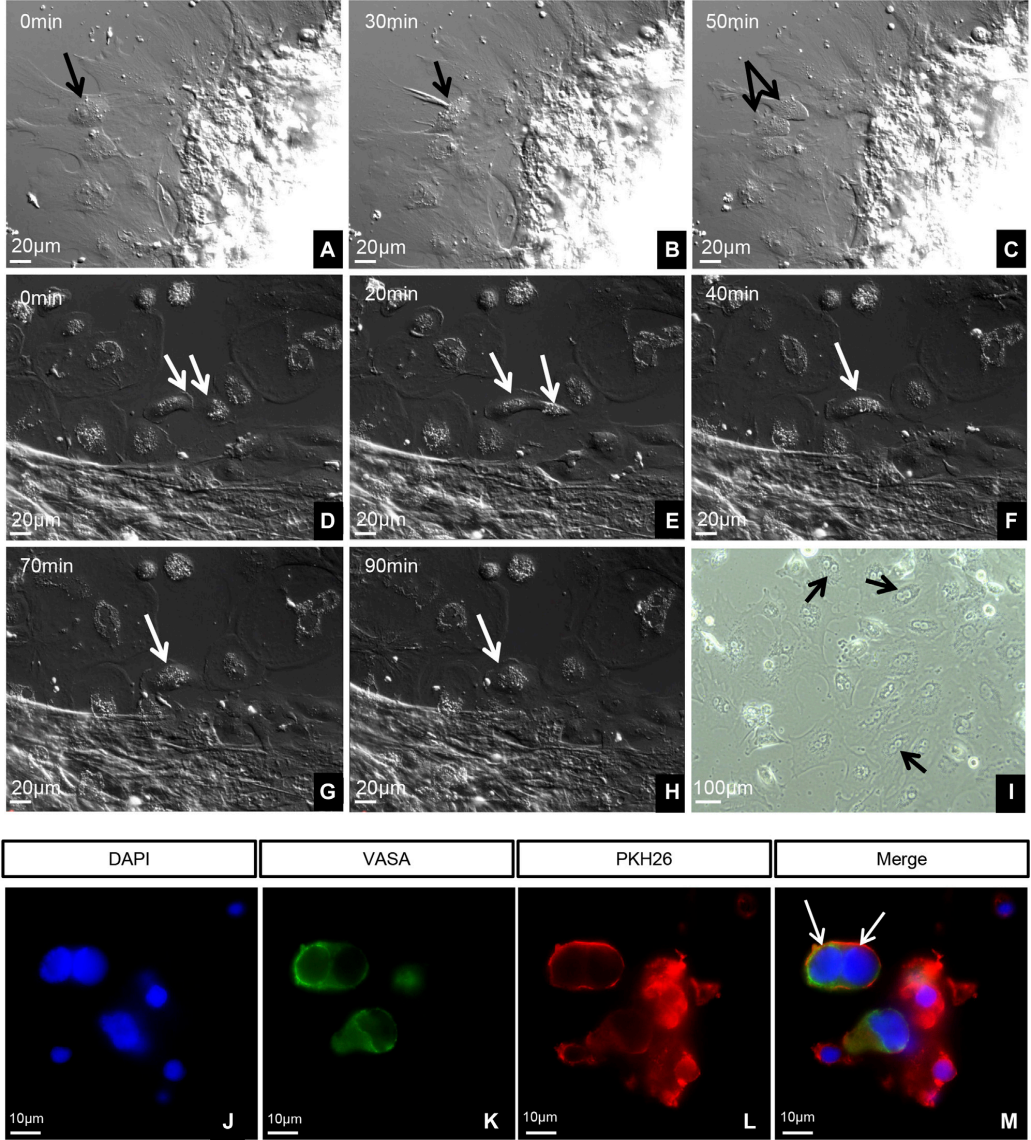
Dynamics and identification of living germ cells. **(A–H)** Time-lapse images taken with a 40 differential interference contrast (DIC) microscope. **(I)** Image taken with 10 phase contrast (PH). **(A–C)** Five-month-old ovary tissues cultured in vitro. **(D–I)** Seven-month-old ovary tissues cultured in vitro. Arrows in panels indicate the following features: **(A–C)** Normal mitosis in ovary. **(D)** Two cells before fusion. **(E)** Cytoplasm of two cells fusing. **(F)** Beginning of the fusion of two nuclei. **(G)** Complete fusion of two nuclei. **(H)** Complete fusion of two cells. **(I)** Many fused cells with two nuclei. **(J–M)** Identification of germ cells using VASA. The arrows indicate the germ cells with double nuclei.

At 5 months old, ovary tissues grew well *in vitro*, and only normal mitosis was observed (Figures 3A–C). However, the 7-month-old ovaries exhibited an unusual phenomenon: the gradual fusing of pairs of cells (Figures 3D–H). At this stage, many binucleated cells were observed (Figure 3F). Immunofluorescence labeling of the germ cells (VASA, green fluorescence) showed that the binucleated cells were germ cells (Figure 3J–M).

### Morphological changes in the chromosomes during prophase I and the formation of the synaptonemal complex

**FIGURE 4:**
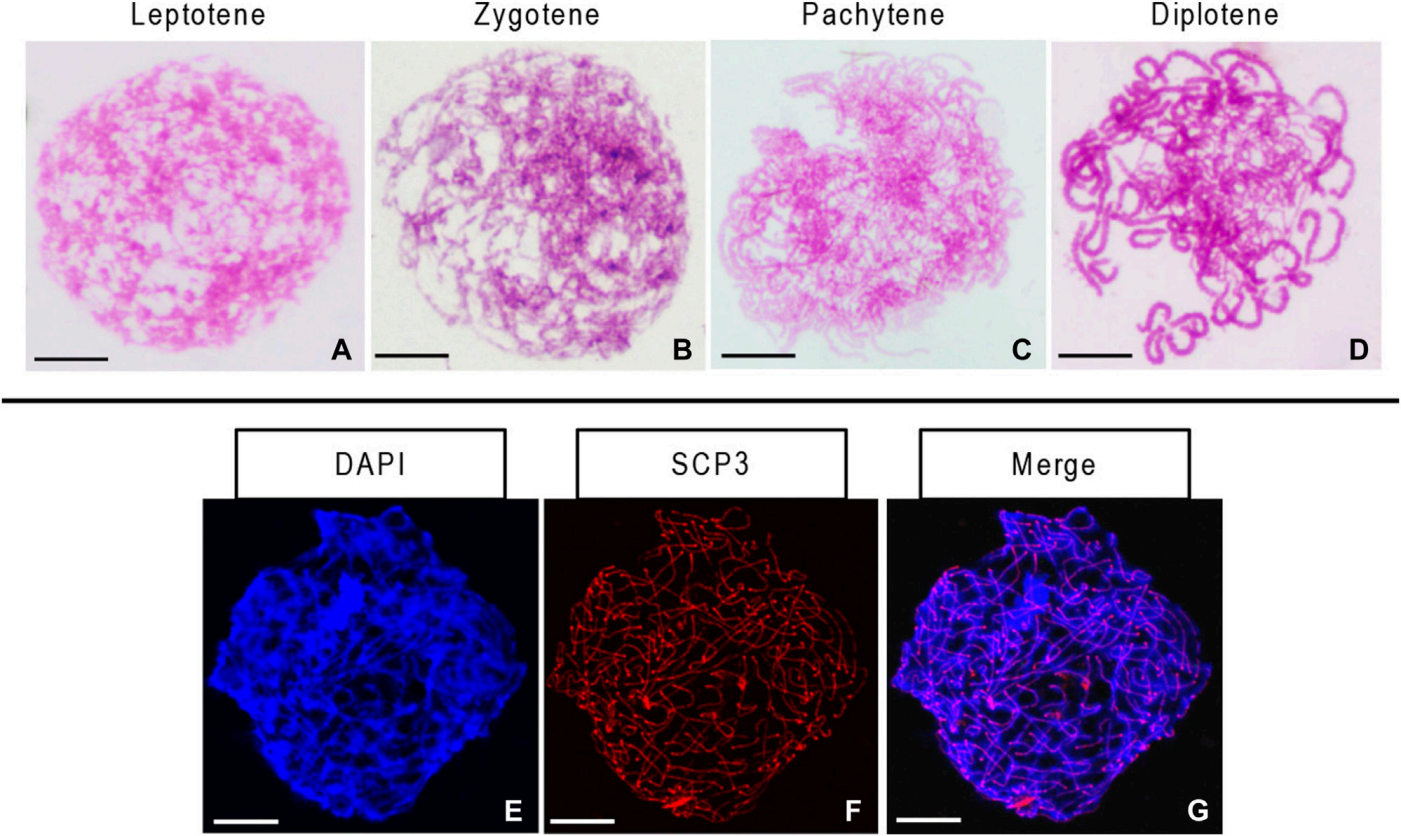
Stages of chromosome meiosis and immunofluorescent detection of synaptonemal complex protein 3 (SCP3) in 10-month-old gynogenetic allodiploid hybrid clone line (GDH) germ cells **(A–D)** Chromosomes at the leptotene, zygotene, pachytene, and diplotene stages **(E–G)** Immunofluorescent detection of SCP3. Scale bars = 20 μm.

We observed several morphological changes in the germ cell chromosomes of the 10-month-old ovary during leptotene, zygotene, pachytene, and diplotene. We found the condensation of chromosomes into a compact structure as filaments in the leptotene stage (Figure 4A). As the homologous chromosomes paired and synapsis progressed, the chromosome thickened gradually and then entered the zygotene and pachytene stages (Figures 4B, C). During the diplotene, X-shaped structures formed due to the dissolution of the synaptonemal complex and the separation of the bivalent homologous chromosomes (Figure 4D). We identified the synaptonemal complex protein SCP3 in all chromosomes in the germ cells from the 10-month-old ovary. The synaptonemal complex structure was complete in this stage (Figures 4E–G).

### Homologous chromosome pairing

**FIGURE 5:**
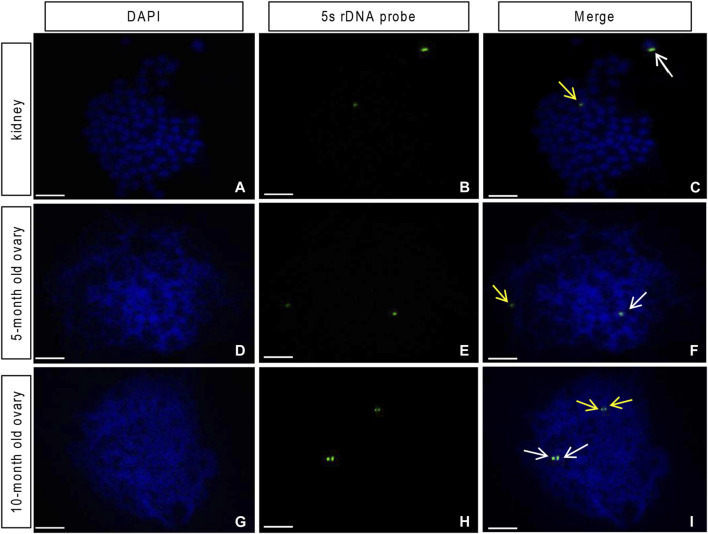
Loci of the 5S rDNA probe from the red crucian carp on the gynogenetic allodiploid hybrid clone line (GDH) chromosomes **(A–C)** Head kidney cell chromosome (expected chromosome number is 100), showing one strong and one weak fluorescent signal **(D–F)** germ cell chromosome of the 5-month-old ovary, showing one strong and one weak fluorescent signal **(G–I)** germ cell chromosome of the 10-month-old ovary, showing paired strong and weak fluorescent signals. Arrows indicate signal points. Scale bar = 10 μm.

Previous studies reported two strong and two weak signals from the 5S rDNA probes in the mitotic metaphase chromosome of the diploid crucian carp (Zhang et al., 2015). By comparison, the common carp showed no such signals (Zhang et al., 2015; Ye et al., 2017). In this study, using the 5S rDNA probe specific to the crucian carp, we identified one strong signal and one weak signal in mitotic metaphase chromosome spreads of both head kidney cells (Figures 5A–C) and oogonia from the 5-month-old ovary of GDH (Figures 5D–F), indicating that these cells possessed one set of crucian carp chromosomes. In contrast, in the chromosomes from the germ cells from the 10-month-old ovary, we observed one pair of strong signals and one pair of weak signals (Figures 5G–I), indicating that the crucian carp chromosomes were doubled and paired at this stage.

### Expression patterns of genes and proteins associated with synapsis and recombination

**FIGURE 6:**
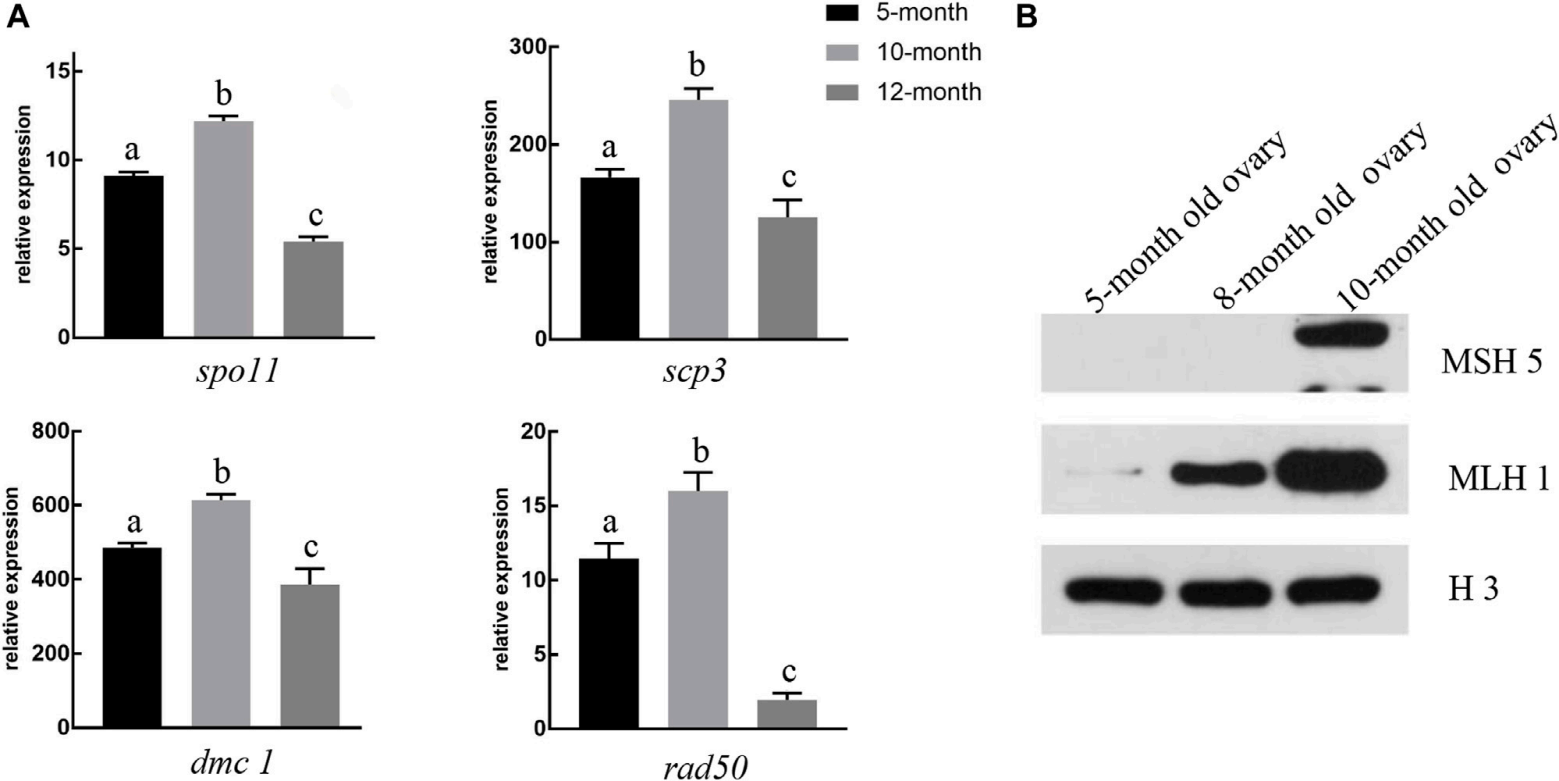
Expression levels of genes and proteins associated with synapsis and recombination **(A)** In 10-month-old gynogenetic allodiploid hybrid clone line (GDH) ovaries, the expression levels of *spo11*, *scp3*, *dmc1,* and *rad50* were highest in five- and 12-month-old ovaries. Different lowercase letters above the bars indicate significant differences (*p* < 0.05) **(B)** Representative Western blot image showing the relative accumulation of recombinant proteins MSH5 and MLH1 in 5-, 8-, and 10-month-old GDH ovaries. H3 was used as the positive control.

To investigate the molecular mechanisms underlying the formation of the synaptonemal complex (SC) during GDH meiosis I, we characterized the expression patterns of four genes and two proteins associated with the synapsis and recombination in ovaries of different ages. All four genes (*spo11*, *scp3*, *dmc1,* and *rad50*) were significantly upregulated in the 10-month-old ovary as compared to both the 5-month-old ovary and the 12-month-old ovary; *spo11* and *rad50* were also significantly upregulated in the 5-month-old ovary as compared to the 12-month-old ovary (Figure 6A).

Low levels of MLH1 expression were detected in the 5-month-old GDH ovary (Figure 6B). The expression levels of MLH1 increased both in the 8-month-old and the 10-month-old ovary, while MSH5 was only detected in the 10-month-old ovary (Figure 6B).

## Discussion

### Reproductive characteristics of hybrid fish

During gametogenesis, the synapsis of homologous chromosomes is an important guarantee of genetic lineage stability and variability. Allodiploids are generally sterile due to the absence of homologous chromosomes. However, fish reproductive strategies are highly plastic: when genetic factors, such as hybridization incompatibility, inhibit normal production and development of haploid gametes (genetic material halved), reproductive modes often shift to adapt. For example, fish may produce unreduced gametes (Zhang et al., 2005).

In general, diploid fish produce haploid gametes. However, the gynogenetic allodiploid hybrid clonal line (GDH), derived from hybridization of crucian carp and common carp, produces diploid eggs. Once activated by UV-irradiated sperm, these diploid eggs develop into the next-generation of diploid gynogens without chromosome doubling (Liu et al., 2004). If the sperm are not irradiated, the diploid eggs are fertilized, forming triploid offspring (Liu et al., 2004). Hence, it is easy to distinguish between the gynogenic offspring and the hybrid offspring. In addition, the results of RAPD assay and the microsatellite analysis showed that GDH presented lower level of polymorphism than the original female parent allotetraploids, suggesting that gynogenesis increased the genetic homogeneity of GDH. Consistent with this, the RAPD results indicated the average genetic similarity coefficient between GDH_1_ and the corresponding female parent was 0.97, whereas that between GDH_1_ and the corresponding male parent (which provided the UV-irradiated sperm for egg activation) was only 0.60 (Yan et al., 2005). These findings implied that GDH was obtained from gynogenesis. Furthermore, GDH egg diameter is 0.17 cm, identical to that of diploid eggs stripped from allotetraploids, and fertilization cytology showed the normal expulsion of the second polar body during gynogenesis (Zhang et al., 2005). This indicated that the eggs produced by GDH are diploid.

There have been other reports of this particular type of reproduction. For example, female F_1_ hybrids of crucian carp (*Carassius auratus gibelio*) and common carp (*Cyprinus carpio* L.) produce diploid eggs (Cherfas et al., 2010), as do female hybrid Medaka (*Oryzias latipes* crossed with *O. curvinotus*) (Shimizu et al., 2000), and female hybrids of Thai walking catfish (*Clarias macrocephalus*) and African catfish (*Clarias gariepinus*) (Na-Nakorn et al., 2004). The reproductive patterns of these hybrid fish have altered due to dramatic changes in the genetic makeup of their heterologous chromosomes.

The sterility of many interspecific hybrids arises from failures during meiosis: because homologous chromosomes must align precisely to form synaptonemal complexes and exchange genetic material, accurate chromosome synapsis, recombination, and segregation during meiosis is essential for the production of normal gametes (Pawlowski et al., 2004; Park et al., 2020). In many interspecific hybrids, the allodiploid chromosomes behave independently during meiosis I, with few interactions between paternal and maternal chromosomes, and thus form univalent chromosomes before segregation (Park et al., 2020). In the absence of synaptonemal complexes between homologous chromosome pairs, or when complexes are formed between multiple and/or non-homologous chromosomes, the chromosomes segregate abnormally, resulting in aneuploid gamete formation and sterility (Martinez-Perez and Colaiácovo, 2009; Park et al., 2020). However, to reduce the frequency of sterile offspring from interspecific crosses, some fish have developed special reproductive modes, including gynogenesis, androgenesis, and the formation of unreduced gametes. Previous studies have shown that fertile allodiploid or triploid hybrids are produced in crosses involving crucian carp, salmon, trout, loach, and silver carp (Benfey and Sutterlin, 1984; Galbreath and Thorgaard, 1995; Tiwary et al., 2000; Cherfas et al., 2010; Kuroda et al., 2019; Knytl et al., 2022). GDH is an intergeneric allodiploid with two sets of chromosomes, one from each parent (crucian carp and common carp) (Wang et al., 2016). Although intergeneric allodiploids are often sterile, GDH is not because this hybrid produces diploid eggs without reduction (Liu et al., 2007). If either the first or second polar body is not extruded, it would lead to the production of gametes with unreduced ploidy level. But previous studies have shown that GDH germ cells undergo meiosis; the discharge of the first and second poles was observed, and diploid eggs with the same number of chromosomes as the somatic cells were produced (Zhang et al., 2005). However, it is unclear whether and how the homologous chromosomes undergo correct synapsis, recombination, and segregation. In order to help address this knowledge gap and to better understand the mechanisms underlying hybrid reproduction, we studied chromosome behavior during hybrid oogenesis.

### Formation of the synaptic complex

During the early development of the GDH ovary (<5-month-old), chromatin distribution was uniform in the oogonia, and normal mitosis was observed in ovarian tissues grown *in vitro*. At this stage, the FISH analysis showed that each oogonium possessed one set of crucian carp chromosomes and one set of common carp chromosomes. This indicated that the GDH germ cells with heterogeneic genetic compositions had normal mitotic proliferation. Both observations of chromosome behavior and expression profiling of related genes and proteins showed that meiosis did not occur in the 5-month-old GDH ovaries. In general, 5-month-old crucian carp and common carp males produce sperm, while the 5-month-old crucian carp and common carp female gametes have entered the diplotene phase of meiosis I.

We observed an obvious bouquet phase in the 10-month-old GDH ovary, with the post-leptotene chromosomes gathering in one region of the nucleus. The telomeres of the chromosomes are attached to a relatively small area (segment) of the nuclear envelope *via* a specialized conical thickening; this polarized chromosomal array is known as the bouquet (Harper et al., 2004). Bouquet formation restricts chromosome mobility at the telomeres, and telomere-led chromosome oscillation promotes pairing of homologous regions followed by synapsis and meiotic recombination between homologues (Moiseeva et al., 2017). This result was consistent with our FISH analysis, in which signals corresponding to homologous chromosomes were paired in the GDH germ cell. In addition, during the bouquet stage (10-month-old), proteins and genes associated with synapsis and recombination were significantly upregulated, indicating that synapsis and recombination were progressing in the germ cell.

In general, the pairing failure of heterologous chromosomes in allodiploids will interrupt meiosis. The alleviation of such blockages and the repair of associated defects may be driven by a meiosis checkpoint in GDH.

### Doubling of chromosomes

Meiosis is a complex and delicate process, and most organisms have evolved meiosis-specific checkpoints, such as the pachytene check point, which monitors the fidelity of chromosome synapsis and the repair of DNA damage. Programmed DNA double-strand breaks activate the pachytene check point, which in turn initiates the homologous recombination repair of the double-strand breaks (Bishop et al., 1992; Sym et al., 1993; Chua and Roeder, 1998; Gartner et al., 2000). This checkpoint causes defective meiocytes to self-destruct, thus preventing the generation of defective gametes (Li et al., 2009). In organisms with a strict pachytene checkpoint, triploid or allodiploid individuals cannot generate gametes, and meiotic cells with pairing or recombination defects are aborted (Li et al., 2009).

Due to the absence of homologous chromosomes in the GDH germ cell at the early development stage, the pachytene checkpoint inhibited the meiosis process. This may explain why synapsis was not observed in the 5-month-old germ cells. Diploid eggs were finally obtained from two-year-old GDH. The abnormalities in chromosomes recombination and synapsis were resolved by doubling the genetic material. We observed binucleated cells in the microstructure of the GDH ovary. The binucleated cells formed by cell fusion, as was demonstrated by *in vitro* cell cultures. Tissue immunofluorescence with VASA showed that the binucleated cells were germ cells. In GDH, genetic material was doubled before meiosis to ensure that the next homologous synapsis proceeded normally. This may explain the unusual length of early ovarian development in GDH. In general, crucian carp reach sexual maturity at 1 year of age, and early ovarian development (before the growth phase) is relatively short, lasting about 2 months (Wang et al., 2016). In GDH, however, this phase of ovarian development lasted more than 10 months and was concomitant with cell fusion activity (Wang et al., 2016). Hybrid sterility is often caused by the abnormal pairing of homologous chromosomes during meiosis. Correct homologous syndication is necessary for gamete formation in GDH.

In conclusion, the absence of chromosomal pairing and synaptonemal complexes in GDH during early development (<5 months old) suggested that synapsis and recombination were suppressed between non-homologous chromosomes in this hybrid. In synthesized allopolyploids such as GDH, it is necessary to double the genetic material to form pairable homologous chromosomes for correct meiotic progression, which is important for the formation of viable gametes and reproductive success in the hybrid progeny (Park et al., 2020). In GDH, germ cell chromosomes doubled through fusion; subsequent pairing and synapsis with homologous chromosomes proceeded normally. Further in-depth studies of fish reproductive strategies are required to identify the underlying regulatory mechanisms. Such studies will also help to provide a theoretical basis for optimized fish breeding programs, taking advantage of polyploid formation.

## Data Availability

The original contributions presented in the study are included in the article/supplementary material, further inquiries can be directed to the corresponding author.
